# Attitudes of serodiscordant couples towards antiretroviral-based HIV prevention strategies in Kenya: a qualitative study

**DOI:** 10.7448/IAS.17.4.19563

**Published:** 2014-11-02

**Authors:** Nikola Fowler, Paul Arkell, Michael Abouyannis, Catherine James, Lesley Roberts

**Affiliations:** 1 College of Medical and Dental Sciences, University of Birmingham, Birmingham, UK; 2 Foundation Programme, West Midlands Deanery, Birmingham, UK; 3 Emergency Department, Stanger Provincial Hospital, Stanger, South Africa; 4 Primary Care Clinical Sciences, University of Birmingham, Birmingham, UK

## Abstract

**Introduction:**

Transmission in serodiscordant couples (SDCs) accounts for approximately half of all new HIV infections, both in Kenya and the wider sub-Saharan region [1]. With evidence to suggest inconsistent condom use within this population [2], the World Health Organization has recommended two new methods of HIV prevention for SDCs: Treatment as Prevention (TasP) and Pre-Exposure Prophylaxis (PrEP). However, there has been little research about the attitudes of SDCs towards these strategies [3, 4]; knowledge that is paramount for successfully predicting the acceptability and efficacy of each method, as well as for informing decisions regarding HIV policy changes in Kenya.

**Methods:**

An exploratory, qualitative study was conducted in the Muhoroni constituency of Nyando district, Kenya from January to March 2013. Purposive sampling was predominately used to recruit 21 HIV-positive and 17 HIV-negative individuals in a serodiscordant relationship from four hospitals and health centres. During face-to-face semi-structured interviews, topic guides were used to elicit information about participants’ attitudes and preferences towards TasP and PrEP. Collected data underwent framework analysis, allowing the development of overarching categories, sub-themes and inductive interpretation.

**Results:**

The majority of participants, irrespective of gender and HIV status, found TasP more acceptable than PrEP. A key factor influencing this decision was HIV-negative participants’ limited motivation to take and adhere to antiretrovirals (ARVs), primarily due to a predominantly external health locus of control, a lack of cultural acceptance of prophylactic medication and concerns about side effects. In addition to this, the likely health improvements TasP offers HIV-positive partners, as well as the attitude that the sick individual should be the first to receive HIV medication, also contributed to this conclusion. Issues of risk compensation were raised, with some HIV-negative partners indicating a desire to stop using condoms if ARV-based prevention methods were available.

**Conclusions:**

Findings from the study indicate that TasP may represent a more viable approach to HIV prevention in Kenya than PrEP. Couples’ preferences, however, may differ depending on local attitudes towards prophylaxis and health locus of control.
